# Editorial: Broadening our understanding of the impact of infections on the developing central nervous system - from basic to clinical sciences

**DOI:** 10.3389/fneur.2023.1198518

**Published:** 2023-05-10

**Authors:** Mark A. Travassos, Ursula K. Rohlwink, Elizabeth W. Tucker

**Affiliations:** ^1^Center for Vaccine Development and Global Health, University of Maryland School of Medicine, Baltimore, MD, United States; ^2^Division of Neurosurgery, Department of Surgery and Neuroscience Institute, Faculty of Health Sciences, University of Cape Town, Cape Town, South Africa; ^3^Department of Anesthesiology and Critical Care Medicine, Johns Hopkins University School of Medicine, Baltimore, MD, United States

**Keywords:** tuberculous meningitis, cerebral malaria (CM), central nervous system infection, pediatric infection, pediatric infectious disease, *Listeria* meningoencephalitis, tuberculosis, malaria

“Beth did have the fever... She lay in that heavy stupor, alike unconscious of hope and joy, doubt and danger. It was a piteous sight, the once rosy face so changed and vacant, the once busy hands so weak and wasted, the once smiling lips quite dumb, and the once pretty, well-kept hair scattered rough and tangled on the pillow.”—Louisa May Alcott, *Little Women* (1).

Infection casts a long shadow over the young March sisters in Louisa May Alcott's 19th century novel *Little Women*, particularly as the family witnesses Beth's struggles through fever, delirium, and the persistent effects of illness, eventually passing away years later. Infections of the central nervous system still represent a major global burden of disease in children, as we have a poor understanding of disease processes, optimal treatment, and long-term impact. This is particularly true of neurological manifestations of historically understudied diseases like tuberculosis and malaria. Children are distinct from adults and subject to a changing biological landscape over the course of childhood. The developing brain has unique vulnerabilities depending on the age of the child as the innate and adaptive components of the immune system mature (Singh et al.). Young children are the most vulnerable to disease, particularly those with malnutrition. Importantly, a young child's response to infection depends on features of a host response that are also essential to healthy neurodevelopment (Kim et al.). As a result, infections in the developing brain can have long-lasting sequelae that can persist well after the initial insult. This Research Topic aims to broaden our understanding of infection in the developing central nervous system, to begin to unravel pathophysiology and pathogenesis, to elucidate acute and long-term treatment and monitoring strategies that may improve outcomes, and to address disease burden and impact at a public health level.

A major theme of the work in this Research Topic is how little is known about many of the infections under investigation. Cerebral malaria represents one of the most understudied—but devastating—diseases of children, principally affecting children in rural sub-Saharan Africa, where there is a dearth of healthcare resources. We have only recently begun to identify the malaria proteins critical to this disease (2, 3) and define associated host immune vulnerabilities (4). The landscape of cerebral malaria is changing as new malaria control measures have been rolled out across the African subcontinent, and it is critically important to know if this has resulted in shifts in the incidence of cerebral malaria in the community, as suggested by the study by Coulibaly et al.. Particularly as a novel malaria vaccine is implemented across much of sub-Saharan Africa, we must understand how this could impact the burden of this disease.

Similarly, tuberculous meningitis represents the deadliest form of extrapulmonary tuberculosis. There is a clear need for improved surveillance of tuberculous meningitis (du Preez et al.), as missing this diagnosis has dire consequences for the patient. Schurz et al. has identified possible genetic vulnerabilities that may lead to the development of tuberculous meningitis (Schurz et al.). This study and the genetic approach it described provides a foundation for further investigations of genes that may contribute to host vulnerabilities to tuberculous meningitis. Salih et al. reports on critical factors associated with severe tuberculous meningitis in South African children, including the severity of hyponatremia and low glucose levels in the cerebrospinal fluid. The pathophysiology of tuberculous meningitis must also be investigated further. A metabolomic and proteomic study of tuberculous granulomas has provided insights into the disease processes (Sholeye et al.), but there is a need for further “omics” studies with respect to both focal forms of the disease, like granulomas, and diffuse manifestations seen in tuberculous meningitis.

Cutting-edge approaches such as “omics” studies are crucial to illuminate the underpinnings of other pediatric central nervous system infections. That *Listeria* meningoencephalitis can occur in a previously healthy child (Mo et al.) is unexpected and highlights the need for further epidemiologic and pathophysiologic investigation to uncover potential host vulnerabilities. Genomic, transcriptomic, proteomic, and metabolomic studies of such infections hold the promise of identifying critical factors in disease pathogenesis and host vulnerability.

More than 150 years ago, the specter of childhood infections loomed over Louisa May Alcott's writing. In some respects, little has changed. The consequences of neuroinfection during childhood can be life-long and cumulative. The work in this Research Topic highlights some of the progress that has been made in these conditions, but makes clear the urgent need for more pediatric-specific research in this field. Only then can we make real progress toward developing appropriate treatments for these devastating diseases.

## Author contributions

MAT, UKR, and EWT drafted and edited the manuscript. All authors contributed to the article and approved the submitted version.
